# Neovascular glaucoma after central retinal vein occlusion in pre-existing glaucoma

**DOI:** 10.1186/1471-2415-14-119

**Published:** 2014-10-05

**Authors:** Hsi-Fu Chen, Min-Chi Chen, Chi-Chun Lai, Ling Yeung, Nan-Kai Wang, Henry Shen-Lih Chen, Wan-Chen Ku, Shiu-Chen Wu, Shirley H L Chang, Lan-Hsin Chuang

**Affiliations:** Department of Ophthalmology, Chang Gung Memorial Hospital, 222 Mai-Chin Rd, Keelung, 204 Taiwan; Department of Ophthalmology, Chang Gung Memorial Hospital, Linkuo Medical Center, Taoyuan, Taiwan; Department of Public Health and Biostatistics Consulting Center, School of Medicine, Chang Gung University, Taoyuan, Taiwan; Chang Gung University College of Medicine, Taoyuan, Taiwan

**Keywords:** Neovascular glaucoma, Central retinal vein occlusion with pre-existing glaucoma, Retinal non-perfusion status, Intraocular pressure, Predisposing factors

## Abstract

**Background:**

To determine the outcome of central retinal vein occlusion (CRVO) in pre-existing glaucoma and the predisposing factors of developing neovascular glaucoma (NVG).

**Methods:**

We retrospectively assessed a pre-existing glaucoma CRVO group and a non-glaucoma CRVO group to elucidate the demographics, clinical course and ocular parameters of these two cohorts. Among the pre-existing glaucoma cases, the predisposing factors for the development of NVG were monitored, including the retinal capillary non-perfusion status, intraocular pressure (IOP) and best-corrected visual acuity (BCVA) at presentation.

**Results:**

Of 642 CRVO patients reviewed in this 10-year cohort study, 60 (9.3%) had pre-existing glaucoma at a mean follow-up of 30.8 months, including 28 (4.4%) primary open angle glaucoma (POAG), 27 (4.2%) primary angle closure glaucoma (PACG), and 5 (0.8%) normal tension glaucoma (NTG) cases. Although the presence of glaucoma in the CRVO eyes was not significantly associated with the risk of developing NVG, the incidence of developing NVG in pre-existing glaucoma eyes was significantly higher in the group with IOP greater than 20 mmHg at CRVO presentation (*P* = 0.02, Chi-square test) as well as in the ischemic CRVO group compared to the non-ischemic patients (*P* = 0.005, Fisher’s exact test). Overall, 20% of pre-existing glaucoma patients needed glaucoma surgery after a CRVO event, including 11.7% of patients who developed iris neovascularisation (INV) and 8.3% of patients who developed a high IOP without INV.

**Conclusions:**

Both the retinal non-perfusion status and uncontrolled IOP contribute to NVG in patients with pre-existing glaucoma after CRVO. Following CRVO, glaucoma surgery is necessary for pre-existing glaucoma cases with intractable elevated IOP with or without INV.

## Background

Central retinal vein occlusion (CRVO) is a common retinal vascular disease that leads to visual impairment in elderly patients. Previous studies from Western countries have a high incidence of pre-existing primary open angle glaucoma (POAG) in patients with CRVO [1–3]. However, Asian populations differ from Western populations in that the incidence of primary angle closure glaucoma (PACG) is much higher. The incidence of different types of pre-existing glaucoma, intraocular pressure (IOP) changes and optic neuropathy in CRVO in the Asian population is still unclear.

One-third of non-ischemic CRVO may convert to ischemic CRVO [4]. The major complication of ischemic CRVO is neovascular glaucoma (NVG) [4, 5]. In CRVO patients with pre-existing glaucoma who have developed NVG, severe corneal oedema and recalcitrant high IOP are typically difficult to manage. Therefore, damage to the optic nerve is difficult to avoid, and any residual vision may be threatened. Thus far, it is still unknown whether pre-existing glaucoma plays a role in the development of NVG in CRVO patients. Regardless of treatment with intravitreal injection of anti-vascular endothelial growth factor (VEGF) or corticosteroids, conventional panretinal photocoagulation (PRP) can regress retina neovascularisation in CRVO [6].

Because there is a paucity of evidence on this condition, the aim of this cohort study was to determine the profile of pre-existing glaucoma in CRVO in the East Asian population. Additionally, the secondary aims were to explore the predisposing factors and outcomes for the development of NVG in CRVO patients with pre-existing glaucoma.

## Methods

This was a retrospective and comparative long-term cohort study. Patients were enrolled from Jul 1999 to Dec 2009. This study was performed in accordance with the Helsinki Declaration of 1975 (1983 revision) and was approved by the Institutional Review Board of the Chang Gung Memorial Hospital, Linkuo, Taiwan. All patients diagnosed with CRVO were identified and followed up monthly within one year, and their clinical data were recorded during a chart review.

Demographic characteristics, including gender, age, systemic diseases, the onset of CRVO and preceding eye history, were collected. The general ocular examination, including the best-corrected visual acuity (BVCA), slit-lamp biomicroscope examination, IOP at the first diagnosis of CRVO, indirect ophthalmoscopy, fluorescein angiography findings, or related surgery history, was documented in detail.

In this 10-year CRVO cohort study, the study group was consisted of pre-existing glaucoma cases diagnosed with increased IOP, an optic cup/disc ratio larger than 0.5, and glaucomatous visual field changes. NVG was defined as neovascularisation of the iris with an IOP increase ≥ 21 mmHg. Optic nerve damage progression was defined as an increasing cup/disc ratio. The type of primary glaucoma, gonioscopy findings, optic cup/disc ratio and visual field data were also recorded. Newly diagnosed participants with CRVO were classified as the control group. We excluded patients who received intravitreous injection of anti-VEGF medications or triamcinolone acetonide during the follow-up period. As a proceeding analysis, patients who underwent vitrectomy to clear vitreous haemorrhages, patients who presented with distinct iris neovascularisation or neovascular glaucoma during the first visit or patients with significant diabetic retinopathy observed at short-term follow up (shorter than six months) were also precluded from this study. During the follow-up period, details regarding the procedures were also recorded for patients who underwent panretinal photocoagulation (PRP) or filtration surgery after CRVO.

Data were entered into Excel and were analysed using SPSS software (Statistical Package for Social Sciences). The differences between the non-glaucoma and pre-existing glaucoma groups were evaluated using a chi-square test and a 2-sample *t*-test for categorical and continuous variables, respectively. For the patients with pre-existing glaucoma, the relationships between the development of NVG and ocular parameters were examined using the Fisher’s exact test or Wilcoxon rank sum test. Furthermore, the visual and optic nerve changes between the initial and final stages were assessed using the Wilcoxon signed-rank test. In this study, the Snellen visual acuity was converted to the logarithm of the minimum angle of resolution (logMAR) units for analysis. A *p* value of less than 0.05 was considered significant.

## Results

Overall, 642 patients with CRVO were reviewed in this 10-year cohort study. There were 60 eyes from 60 patients who had a history of pre-existing glaucoma before the onset of CRVO. The incidence of pre-existing glaucoma in CRVO was 60/642 (9.3%). Regarding the various types of pre-existing glaucomas, there were 28 primary open angle glaucoma (POAG) cases, 27 primary angle closure glaucoma (PACG) cases and 5 normal tension glaucoma (NTG) cases in this study. A total of 321 patients were excluded from this study due to vitrectomy surgery or intravitreal injections of triamcinolone acetonide or anti-VEGF. To compare the characteristics, 60 pre-existing glaucoma cases were categorised as the study group, and 261 non-glaucoma cases were categorised as the control group.

The demographics, clinical characteristics and baseline ocular parameters are listed in Table 1. In the pre-existing glaucoma group, the age of CRVO onset was significantly older than the control group (57.3 vs. 67.2 years, *p*<0.001). The prevalence of males was much higher than that of females in both groups. When comparing the ocular parameters, the cup/disc (C/D) ratio was substantially larger in the study group compared to the control group. According to the FAG finding, the proportion of ischemic type CRVO was higher in the pre-existing glaucoma group than in the control group (39.8% VS 45%), although not significantly (*p*=0.46). Moreover, during CRVO diagnosis, approximately one half (48.3%) of the pre-existing glaucoma participants exhibited a higher increase in the IOP compared to the control group (21.7±9.2 vs. 13.9±4.8 mmHg, *p*<0.001). However, the vision (BVCA) of both groups at the time of diagnosis was similar. There was no significant difference in the proportion of ischemic type CRVO between the two groups (Table 1). In general, 20.3% (53/261) of the non-pre-existing glaucoma cases and 31.7% (19/60) of the pre-existing glaucoma cases developed NVG.(*p*=0.06) Furthermore, the incidence of NVG occurred within 6 months for ischemic CRVO was100% (19/19) of cases in the pre-existing glaucoma group and 81.18% (43/53) of cases in the control group (*p*= 0.05).

**Table 1:** **Demographic and ocular characteristics of pre-existing glaucoma in central retinal vein occlusion (CRVO)**

	Non-glaucoma	Pre-existing glaucoma	***p value*** ^†^
**Case number**	261	60	
**Gender** (female:male)	102: 159	25: 35	0.712
**Age** (mean±SD)	57.3±17.5	67.2±14.9	<0.001
**Systemic disease**			
DM	71 (27.2%)	14 (23.3%)	0.540
HTN	131 (50.2%)	29 (48.3%)	0.795
**CRVO onset (months)** (mean±SD)	1.6±3.8	1.7±3.6	0.832
**Perfusion status**			0.464
Non-ischemic	157 (60.2%)	33 (55%)	
Ischemic	104 (39.8%)	27 (45% )	
**C/D ratio** (mean±SD)	0.42±0.09	0.72±0.16	<0.001
**IOP at presentation** (mmHg) (mean±SD)	13.9±4.8	21.7±9.2	<0.001
**BCVA** (logMAR) (mean±SD)	1.46±0.85	1.69±0.84	0.053
**PRP during follow up**	107 (41%)	22 (36.7%)	0.537
**NVG during follow up**	53 (20.3%)	19(31.7%)	0.057

Abbreviations: *BCVA* best-corrected visual acuity, *C/D* cup-to-disc, *DM* diabetes mellitus, *HTN* hypertension, *IOP* intraocular pressure, *log MAR* logarithm of the minimal angle of resolution, and *SD* standard deviation.

^†^ *p* values were calculated using the chi-square test and 2-sample t-test for categorical and continuous variables, respectively.

It is well known that the major complication after the onset of CRVO is the development of NVG, and IOP is typically intractable to medical treatment. To further identify the predisposing factors among the ocular parameters that contribute to the development of NVG in the study group, at the first visit, the IOP, C/D, and retinal nonperfusion status were obtained (Table 2). In the univariate analysis of the development of NVG, there was a significant difference in the mean IOP at the time of presentation between NVG within 0.5 yr and non-NVG within 0.5 yr (29.2±10.1 vs. 19.8±8.29, *P*=0.007, Wilcoxon rank sum test). Additionally, when the IOP was dichotomised as >20 or≦20 mmHg, 10 out of 32 (31.3%) patients with IOP >20 mmHg and 2 out of 28 (7.1%) with IOP ≦20 mmHg developed NVG within 0.5 yr, respectively (Chi-square test, *P*=0.020). Considering another fundamental parameter in the pre-existing glaucoma group that is associated with NVG development, a majority (93.3%) of the patients had moderate to severe cupping upon the diagnosis of CRVO. The average C/D upon the presentation of CRVO was 0.72 in the study group. A high incidence of a large C/D ratio was found as 38.3% of the patients in the study group had moderate cupping (0.5≦C/D<0.8) and 55% had severe cupping (≧0.8). For pre-existing glaucoma patients, there was a marginal significant difference in the C/D ratio between NVG within 0.5 yr and non-NVG within 0.5 yr [0.78±0.17, (range: 0.4-0.9) vs. 0.70±0.16, median 0.75 (range: 0.4-0.9) using the Wilcoxon rank sum test (*P*=0.053)]. During the study period of 30.8 months, C/D increased to subtotal cupping in 37 out of 60 (61.7%) cases, indicating that the RNFL continued to thin, which is a serious consequence after retinal haemorrhage is resolved. Furthermore, the incidence of developing NVG was significantly higher in the pre-existing glaucoma and control group patients with ischemic type CRVO compared to non-ischemic type CRVO.(*p*=0.005 and *p*<0.001, Fisher’s exact test).

**Table 2:** **NVG within 0.5 yr after CRVO in pre-existing glaucoma**

	NVG within 0.5 yr	Non-NVG within 0.5yr	***p value***
**C/D ratio at presentation** (mean±SD)	0.78±0.17	0.70 ±0.16	0.053
**Retinal perfusion status**			0.005*
Non-ischemic	5	28	
Ischemic	14	13	
**Mean IOP at presentation** (mmHg) (mean±SD)	29.2±10.1	19.8±8.29	0.007**
IOP > 20 mmHg at presentation	10	22	0.020***
IOP ≦ 20 mmHg at presentation	2	26	

*Fisher’s exact test; **Wilcoxon rank sum test; and ***Chi-square test.

Accordingly, panretinal photocoagulation (PRP) was suggested in cases where neovascularisation of the iris occurred at follow-up. Nevertheless, there were 2 PACG cases with an initially normal IOP, which spiked after PRP was performed, and IOP stabilised with additional medication. Seventy-five percent of pre-existing glaucoma patients were regularly using anti-glaucoma agents before the first CRVO event. Among 27 pre-existing PACG patients, 9 patients had received laser iridotomy. Surgical interventions for NVG patients, including filtration surgery and transscleral cyclophotocoagulation, are summarised in Table 3. For patients with end stage glaucoma and poor vision, the type of surgery performed (trabeculectomy with mitomycin C or transscleral cyclophotocoagulation) depended on the physician’s preference and patients’ agreement. An intractable IOP increase without INV was treated with trabeculectomy with or without an antimetabolite agent if useful vision was still present. In this retrospective cohort study, 12 cases (20%) needed surgical intervention to control IOP during the long-term follow up. The two major causes of surgery were uncontrolled IOP in cases that developed NVG (11.7%) and intractable IOP without iris NV (8.3%). It is worth noting that there were some pre-existing glaucoma cases that flared up with high IOP without INV, and medical intervention was not sufficient thereafter.

**Table 3:** **The management of various types of pre-existing glaucoma in central retinal vein occlusion (CRVO)**

	POAG	PACG	NTG	Overall incidence
**Case number**	28	27	5	
**Prior use of antiglaucomaagents**	26 (92.9%)	16 (26.7%)	3 (60%)	75%
**Mean deviation (dB)**	-15.07	-13.02	-16.21	-14.24
**Trabe/TSCP for NVG**	1/3 (14.3%)	1/2 (11.1%)	0	11.7%
**Trabe for elevated IOP (w/o INV)**	2 (7.1%)	3 (11.1%)	0	8.3%

Abbreviations: *PACG* primary angle closure glaucoma, *POAG* primary open angle glaucoma, *PRP* pan-retinal photocoagulation, *Trabe* trabeculectomy, *TSCP* transscleralcyclophotocoagulation, and *w/o INV* without iris neovascularisation.

Due to the retrospective nature of this study, the exact time point of conversion from the non-ischemic to ischemic type is difficult to detect. For example, a case of NTG with a BCVA of 0.6 with non-ischemic CRVO at presentation converted to ischemic CRVO that was subsequently complicated with NVG. This patient then hesitated PRP, and vision decreased to light sense negative. According to the records, 5 out of 33 non-ischemic cases in the pre-existing glaucoma group (15.1%) developed NVG, and ischemic development was noted as possible for future development during the study period.

In general, vision did not significantly improve after conventional glaucoma management up to the 31-month follow-up. In patients with pre-existing glaucoma, vision deteriorated from an initial BCVA log MAR 1.69 (Snellen 0.02) to a final BCVA log MAR1.84 (Snellen 0.014). Because there are three types of pre-existing glaucoma, we analysed the visual outcome based on the mean BCVA and cup disc ratio change (Table 4). As indicated above and in Table 1, the C/D ratio for pre-existing glaucoma cases at CRVO presentation was larger than the control group (0.72 vs. 0.42). When the initial and final ratios were compared, the C/D ratio significantly increased in the overall, POAG, and PACG subgroups. This result demonstrates that the retinal nerve fibre layer defect continued to progress after CRVO, which may be associated with further deterioration of vision for these pre-existing glaucoma cases.

**Table 4:** **Visual and optic nerve changesin various pre-existing glaucoma types**

	POAG	PACG	NTG	Overall
**Case number**	28	27	5	
**IOP at presentation** (mmHg)	24.3±10.0	19.7±7.4	18.6±11.3	21.7±9.2
**Baseline BCVA** (Snellen)	0.027	0.015	0.023	0.02
**Final BCVA** (Snellen)	0.018	0.013	0.005	0.014
**Cup-to-disc ratio**				
**Initial** (mean±SD)	0.69±0.17	0.72±0.16	0.88±0.04	0.72±0.16
**Final** (mean±SD)	0.75±0.16	0.77±0.14	0.88±0.04	0.77±0.15
***p*** **value** ^**†**^	0.01	0.036		0.001
**Follow-up** (months) (mean±SD)	30.1±26.9	32.3±32.8	25.8±22.7	30.8±29.1

Abbreviations: *BCVA* best-corrected visual acuity, *NVG* neovascular glaucoma, *PACG* primary angle closure glaucoma, *POAG* primary open angle glaucoma, and *SD* standard deviation.

^†^Changes betweenthe initial and final cup-to-disc ratio were examined using the Wilcoxon signed-rank test.

## Discussion

Glaucoma is one of the known risk factors for CRVO [1, 2, 7, 8]. A major complication of CRVO is the development of NVG, which can lead to severe visual impairment. Pre-existing glaucoma is seldom included in CRVO studies in Asian populations where the prevalence of PACG is higher than in the Western countries. Ethnicity may play an important role when evaluating ocular diseases related to glaucoma.

In this 10-year cohort study, the incidence of pre-existing glaucoma in CRVO was similar to previous studies [7, 9]. Recently, Michaelides and Foster also suggested that angle-closure may be associated with retinal vein occlusions in retrospective case series with diverse ethnicity [10]. In contrast, Hayreh et al. concluded that compartment syndrome contradicts the concept of playing a role in RVO [11]. In this study, it is notable that the incidence of PACG was approximately equal to POAG in CRVO. Regarding demographic and ocular parameters, CRVO was more prevalent in the elderly and middle to late stage glaucoma cases with large cupping. Although we do not have exfoliative glaucoma cases in our study, Prata et al. manifested that retinal vascular occlusion in patients with exfoliation syndrome occurs most frequently in the affected or more severely affected eye [12]. This is in agreement with the consensus that large cupping in glaucoma patients is vulnerable to CRVO [3, 12]. Moreover, the incidence of the ischemic CRVO type in pre-existing glaucoma was similar to that in the non-glaucoma population.

Beaumont et al. found that retinal venous occlusion at the optic cup has a higher IOP, C/D ratio and prevalence of POAG [3]. In contrast, Hayreh et al.compared CRVO patients and found that even without glaucoma medication, patients with pre-existing glaucoma had a lower IOP in the lesion eye compared to patients without pre-existing glaucoma [13, 14]. Cappin et al. found that the IOP was 4 mmHg lower in the CRVO eye compared to the other eye in glaucoma patients. By iris angiography, they also found that the vessel of the iris was relatively dilated in CRVO eyes [15]. However, in the Taiwanese population, 46.7% and 45% of patients in the pre-existing glaucoma group were POAG and PACG, respectively. The IOP at CRVO presentation was higher in the pre-existing glaucoma group (mean: 21.7 mmHg) compared to the non-pre-existing glaucoma group (13.9 mmHg). Because the mean diagnosed time was approximately 1.5 months in this study, it is unknown whether the IOP increase occurred before or after CRVO. The exact mechanism of IOP change after CRVO in different types of glaucoma requires further evaluation.

According to an evidence-based study on the natural course of CRVO, the proportion of non-ischemic cases that converted to ischemic cases was as high as 30% in three years [4]. The ischemic type CRVO can lead to NVG in 20% of patients [4]. In our study, for ischemic CRVO, there were more cases with NVG within 6 months in the pre-existing glaucoma group than the control group, but the number of cases was similar after long-term follow up. It is important to understand the other predisposing factors that contribute to the development of NVG in cases with primary glaucoma. We found that in patients with pre-existing glaucoma, the formation of NVG within 0.5 yr was significantly, positively correlated with IOP at presentation and marginally correlated with the C/D ratio at presentation. Due to the retrospective nature of this study, it is difficult to determine the exact IOP changes at the onset of CRVO. However, higher IOP or poor IOP control could theoretically compromise blood perfusion and increase the risk of neovascularisation of the iris. Therefore, it is important to periodically measure the IOP and control IOP after a CRVO event.

There was a similar incidence of the conventional management of PRP among the two groups, which suggests that pre-existing glaucoma is not prone to rubeosis iridis. According to the results of this study, the incidence of neovascular glaucoma with increased IOP was lower than the incidence of PRP performed for cases of rubeosis iridis. Although PRP was effective in eliminating ocular neovascularisation in CRVO in this study, Hayreh et al. revealed marked peripheral visual field loss after laser treatment [16]. One of the drawbacks of this retrospective study was the absence of visual field data collected after a CRVO event from; as a result, we could not conclude anything about the change in the visual field. Furthermore, a relatively high proportion (approximately 20%) of pre-existing glaucoma patients underwent glaucoma surgery after a CRVO event. This is the first study to address the consequence of two major causes of glaucoma surgery, NVG or persistent elevated IOP without ocular neovascularisation. The pathogenesis of intractable increased IOP without iris neovascularisation after CRVO is not clear. In previous reports, the levels of vascular endothelial growth factor were increased in the aqueous humour of glaucoma cases [17]. After a CRVO event, reduced blood flow in the retinal capillaries leads to hypoxia, upregulation and release of VEGF and ocular neovascularisation. Intractable IOP may be secondary to hypoxic and inflammatory changes after CRVO, all of which aggregated in the pre-existing glaucoma cases even though ocular neovascularisation did not develop.

According to an epidemiology study, the severity of diabetic retinopathy and performance of PRP do not correlate with progressive enlargement of cupping [18]. In contrast, this study found that the ischemic change of the retina after CRVO in pre-existing POAG and PACG revealed a retinal nerve fibre layer defect, which progressed throughout the study period. Although with the limitation of the study that different clinicians observed the cupping, this study illustrates the change in the neuroretinal rim in CRVO with pre-existing glaucoma and may partially explain the reason for progressive deteriorated vision in these patients. Generally speaking, vision progressively deteriorated during the long-term follow up in this cohort study.

## Conclusions

The distribution of pre-existing glaucoma in CRVO in the Taiwanese population is different from Western countries. Identifying the profile of pre-existing glaucoma in CRVO is fundamental to further identify effective management strategies. Both the retinal non-perfusion status and uncontrolled IOP contribute to NVG in patients with pre-existing glaucoma after CRVO. Physicians should be aware of the increased IOP after CRVO during follow-up, and glaucoma surgery is necessary for pre-existing glaucoma cases with intractable elevated IOP with or without INV. For such underlying glaucoma diseases associated with glaucomatous optic nerve atrophy, prompt beneficial therapy to prevent complications and preserve vision requires further study.
